# Postprandial ghrelin suppression is exaggerated following major surgery; implications for nutritional recovery

**DOI:** 10.1186/1743-7075-4-20

**Published:** 2007-10-08

**Authors:** Mohsen Nematy, Audrey E Brynes, Philip I Hornick, Michael Patterson, Mohammad A Ghatei, Stephen R Bloom, Stephen J Brett, Gary S Frost

**Affiliations:** 1 Nutrition and Dietetic Research Group, Hammersmith Hospital, Imperial College London, W12 0HS UK; 2 Faculty of Medicine, Mashad University of Medical Sciences, Mashad, Iran; 3 Cardiothoracic Surgery, NHLI, Hammersmith Hospital, Imperial College London, W12 0HS, UK; 4 Department of Metabolic Medicine, Imperial College Faculty of Medicine, London Hammersmith Hospital, London W12 ONN, UK; 5 Division of Surgery, Anaesthetics and Intensive Care, Hammersmith Hospital, Imperial College London, W12 0HS, UK; 6 School of Biomedical and Molecular Sciences, University of Surrey, Guildford, Surrey, GU2 7XH, UK

## Abstract

Meeting patients' nutritional requirements and preventing malnutrition is a challenge following major surgical procedures. The role of ghrelin in nutritional recovery after non-gastrointestinal major surgery is unknown. We used coronary artery bypass grafting (CABG) as an example of anticipated good recovery post major surgery.

Seventeen patients undergoing CABG (mean ± SEM: 70.1 ± 2.2 yrs, BMI 29.1 ± 1.4 kg/m^2^, 15 male) underwent fasting and postprandial (45 mins after standard test breakfast) blood sampling pre-operatively (day 0), post-operatively (day 6) and at follow-up (day 40). Changes in food intake, biochemical and anthropometric markers of nutritional status were recorded. A comparison was made to 17 matched healthy controls (70.6 ± 2.3 yrs, BMI 28.4 ± 1.3 kg/m^2^).

We observed significantly increased post-operative and follow-up fasting ghrelin concentrations compared with pre-operatively (pre-op. 402 ± 42 pmol/L vs post-op. 642 ± 97 pmol/L vs follow-up 603 ± 94 pmol/L) (ANOVA *p *< 0.05). Significantly exaggerated postprandial suppression of ghrelin was seen postoperatively and continued until follow-up (Δ pre-op. 10 ± 51 pmol/L vs Δ post-op. -152 ± 43 pmol/L vs Δ follow-up -159 ± 65 pmol/L, *p *< 0.05). This was associated with a 50% reduction in food intake {post-op. 4.5 ± 0.5 MJ/D (1076 ± 120 kcal/D) compared with estimated requirements 9.9 ± 0.5 MJ/D (2366 ± 120 kcal/D)}, leading to a 4% weight loss and a 5% reduction in muscle arm circumference loss over length of follow up.

Our data support the hypothesis that prolonged changes in fasting and postprandial plasma ghrelin concentrations are associated with impaired nutritional recovery after CABG. These findings reinforce the need to investigate ghrelin in other patients groups undergoing major surgery.

## Findings

Malnutrition remains a largely unrecognised problem in hospitalised patients [1]. Moreover delayed nutritional recovery after routine surgery is also under-appreciated.

Peptide hormones released from the gut have been reported to affect appetite and may play a role in the altered food consumption of patients [2]. Ghrelin, a 28 amino acid peptide, is produced by the stomach, regulates the initiation of feeding, and its level is highest in the fasting state, falling within one hour of a meal [3].

The dynamic response of this hormone in patients who undergo major surgery, for example CABG is unknown. In this study we hypothesised that the driving mechanism for decreased energy intake and weight loss in surgical patients may be abnormal ghrelin concentrations.

We undertook a prospective clinical cohort study in the Department of Cardiothoracic Surgery, Hammersmith Hospital, London UK over a 6 month period. Local research ethics committee approval was obtained for the enrolment of both patients and control subjects (05/Q0406/16); written informed consent was also obtained. The study was performed in accordance with the Helsinki Declaration of 1975 as revised in 1983. Inclusion criteria were patients aged greater than 50 years who were scheduled for an elective CABG procedure. Exclusion criteria included those requiring urgent revascularisation, advanced renal failure, heart failure and severe chronic obstructive pulmonary disease (COPD) with weight loss. Healthy control volunteers matched for age and body mass index (BMI) were recruited by local advertisement.

All CABG patients underwent fasting and postprandial (45 mins after the start of standard test breakfast) blood sampling pre-operatively (day 0), post-operatively (day 6) and at follow up (day 40). The vulnerability of CABG patients, blood loss postoperatively, and ethical issues restricted us to just one postprandial blood sample. The 45 mins time point was used as this has previously been shown to represent the marked nadir in ghrelin [4-6].

The standard test breakfast consisted of 2.76 MJ (660 kcal) (17% protein, 54% carbohydrate and 29% fat) (Ensure Plus, Abbott Nutrition). 15/17 consumed all of the test breakfast on each occasion, 2 patients consumed 75%. Control subjects visited the hospital on 3 separate occasions to undergo fasting blood samples. On the third visit they also consumed a test breakfast as above.

Concentrations of plasma ghrelin immunoreactivity were measured using an established in-house radioimmunoassay as described previously [5]. The assay detected changes of 25 pmol/l of plasma ghrelin with a 95% confidence limit. The intra-and interassay coefficient of variation were 5.5 and 10.1 %, respectively[6]. Appetite, food intake, anthropometric and biochemical indices were collected. Patients and controls were asked to complete a visual analogue scale (VAS) for appetite before and 45 min following each test meal [7]. A 3-day dietary intake was recorded post operation (between days 4–6) and prior to the follow up visit. Estimated energy requirements were used to compare the total energy intake of patients in hospital and at follow-up visits with their estimated nutritional requirements [8,9]. Healthy control subjects also completed a 3-day dietary record. Food intake data were analysed using Dietplan 6 (Forrest Hill Software Ltd, Sussex, UK). Mid arm circumference (MAC) and body weight were assessed pre-operatively, post-operatively, and at follow-up [10,11]. Albumin, protein, C-reactive protein (CRP), and haemoglobin concentrations were extracted from the patient records.

A power calculation based on a previous study using a power of 80% and alpha 5% suggested a sample size of 7 patients matched to 7 controls would be sufficient to show significant changes between patients and control subjects (mean difference 414 pmol/l and standard deviation 248). The data were analysed using SPSS 13.0 for windows (SPSS Science, Apache Software Foundation, Chicago, IL, USA).

All data were checked for normality and presented as mean plus minus standard error of the mean (SEM). Simple or repeated measures analysis of variance (ANOVA) was used for multiple comparison testing; where appropriate, *post hoc *testing was performed using Dunnett and Newmans-Keuls's test respectively. In all cases, p < 0.05 was considered to be statistically significant.

**Figure 1:**
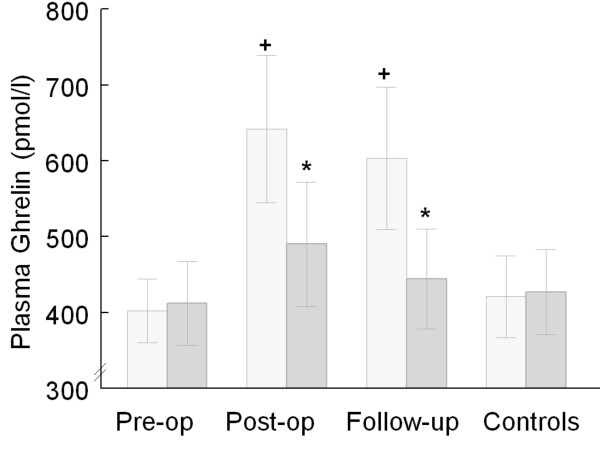
Fasting (light grey bars) and postprandial (dark grey bars) plasma ghrelin concentrations (mean ± SEM) 45 minutes following the start of a test meal in 17 coronary artery bypass grafting patients (CABG) and matched controls, + p < 0.05 fasting postop versus fasting preop & controls, fasting follow-up versus fasting preop & controls ; * p < 0.05 Δ change postop & follow-up versus preop & controls (ANOVA).

Seventeen CABG patients completed the study (mean ± SEM: age 70.1 ± 2.2 yrs, BMI 29.1 ± 1.4 kg/m^2^, 15 male). Patients were compared with matched control volunteers (70.6 ± 2.3 yrs, BMI 28.4 ± 1.3 kg/m^2^, 15 male). There was no difference in fasting plasma ghrelin concentrations between CABG pre-operatively and controls (Figure 1). We observed an increase in post-operative fasting ghrelin concentration compared with pre-operatively. Our data also revealed an exaggerated postprandial suppression of ghrelin concentrations postoperatively and at follow-up, but not pre-operatively nor in control subjects (Figure 1).

Although VAS indicated patients postoperatively reported a higher fasting hunger rating than controls (postop. 38 ± 5 mm vs follow-up 45 ± 8 mm vs controls 23 ± 3 mm *P = 0.02 *and *p = 0.008 *respectively), they had greater postprandial appetite suppression compared to controls. Mean daily energy intake on day 6 was 50% lower in CABG patients compared to their estimated energy requirement {4.5 ± 0.5 MJ/D (1076 ± 120 kcal/D) vs requirements 9.9 ± 0.5 MJ/D (2366 ± 120 kcal/D) *p < 0.001*)} and compared with healthy control subjects {4.5 ± 0.5 MJ/D (1076 ± 120 kcal/D) vs 10.21 ± 0.92 MJ/D (2440 ± 220 kcal/D) *p < 0.001*}. At follow-up patients still did not meet all of their energy requirements; they had a 1.73 MJ (413 kcal) lower daily energy intake than their estimated energy requirements, or 2.17 MJ (519 kcal) lower daily energy intake than control subjects. There was a 4% weight loss and a 5% decrease in MAC from pre-operatively to follow-up, (*p < 0.005 and p < 0.008 respectively*). Post-operatively patients had lower albumin, total protein, and haemoglobin and also higher CRP compared with pre-operatively (*p < 0.0001*). This returned to normal on day 40 at follow-up visit (data not shown).

This is the first report to observe a describe in fasting plasma ghrelin concentrations post-operatively and exaggerated ghrelin suppression following a test meal in CABG patients, despite pre-prandial appetite being preserved. Exogenous ghrelin administration has been shown to increase hunger and food intake [12]. Thus our data are consistent with an increase initial motivation for food postoperatively that was supported by the VAS data. Our data reveal an exaggerated postprandial suppression of ghrelin postoperatively, and at follow-up compared to pre-operatively, which would be expected to be associated with a reduced food intake [5]. The exaggerated ghrelin suppression may be responsible for a reduced drive to eat during meal times, or early satiety. Although ghrelin can be considered as a participant in food intake and body-weight regulation, its role in acute illness is not yet fully understood. It is uncertain whether older individuals with significant underlying medical problems would also adapt appropriately to acute weight loss [13-15].

Results from various recent studies in healthy subjects propose that ghrelin may play a role in appetite regulation. Cummings et al. studied the pattern of plasma ghrelin concentrations over 24 hours. Levels rose by an average of 78% 1–2 h before the onset of each meal, and fell to nadir levels within 1 h after food was first consumed, suggesting that ghrelin concentrations decline quickly after each meal, returning to pre-meal concentrations before the next meal is initiated[3]. Intravenous infusions of ghrelin in healthy human volunteers were also shown to enhance subjectively-rated appetite and to increase energy intake [12]. Furthermore, diurnal rhythms in ghrelin concentrations before and after weight loss were in accord with diurnal rhythms in appetite in humans [16,17]. Test meal studies demonstrated postprandial ghrelin suppression is associated with a reduction in hunger in normal-weight subjects [5,18]. Recent studies on ghrelin shed light on the possible role of this hormone in acute illness. Alterations in ghrelin concentration may have a role to play in appetite and energy intake in acute illness, thus contributing to hospital malnutrition [2]. Previous studies have confirmed the role of ghrelin in meal initiation [3,16]. Results from studies infusing ghrelin in cancer patients [19] and administering subcutaneous ghrelin in dialysis patients with mild to moderate malnutrition [20] suggest ghrelin may be helpful to increase appetite in cachectic or malnourished patients.

In the current study, an acute phase response to surgery was observed during the whole stay. Acute phase response proteins and cytokines release may have affected the gut hormone concentrations. It is not fully understood how changes in acute phase response proteins affect ghrelin concentrations. Further research is required before any dietary interventions, aiming to manipulate ghrelin release can be recommended. It appears that ghrelin plays different roles in several pathologies. Although ghrelin is well-known as a hormone that is associated with negative energy balance, further work in different patient groups might further reveal the role of ghrelin on appetite and food intake.

Particular areas requiring elucidation include the relationship between inflammatory cytokines and gut hormone activity and also the impact of medication, especially antibiotics which affect gut flora, and vasoactive drugs which have an effect on blood flow and motility.

Postprandial ghrelin suppression is a natural response to eating [5], that appears exaggerated after major surgery. In this study patients consumed only half of their energy requirements in the immediate post operative period and had a significant lower daily energy intake at follow-up. Over the 40-day study period they lost 4% body weight and had a 5% reduction in MAC.

Ghrelin, an acylated peptide, stimulates feeding and GH secretion via interactions with the GH secretagogue type 1a receptor (GHS-R1a), the functionally active form of the GHS-R. Ghrelin enhances feeding via the neuronal pathways of neuropeptide Y/AgRP and orexin, which act as orexigenic peptides in the hypothalamus [21]. Central des-acyl ghrelin may activate orexin-expressing neurons, perhaps functioning in feeding regulation through interactions with a target protein distinct from the GHS-R. It is not fully understood how sickness following major surgical procedures affect ghrelin receptors and their functions. Although the mechanism of ghrelin's orexigenic activity is related to the neuropeptide Y (NPY) and agouti-related protein (AgRP) pathways [21], the interaction of ghrelin with other energy-regulating systems is unclear.

In spite of the methodological limitation and being restricted to just one postprandial blood sample (45 mins), we observed an exaggerated ghrelin suppression post-operatively and at follow-up; we might have expected less ghrelin suppression in the CABG patients due to their BMI (29 kg/m^2^) category [5].

Several previous studies have suggested that plasma ghrelin concentration is associated with the short term regulation of appetite and nutritional status [3,5,12].  Recent work by our group showed suppressed baseline concentrations of ghrelin during a 28 day period after admission to intensive care [2]. It is possible that ghrelin, may partly be involved in long term regulation of food intake in parallel with their *short-term *effects in conjunction with several other neural, humeral, and psychological factors that manage this complex process, so called "appetite". This idea is lent support by Cummings *et al*., who have highlighted evidence indicating that ghrelin can be considered as a participant in long-term body-weight regulation [22].

Although this is a first study to assess dynamic responses of ghrelin on a small number of CABG patients, it raises the intriguing possibility that appetite regulatory peptide concentrations may be influenced by surgical procedures, thus contributing to continuing reduced daily energy intake during the recovery period. Further studies are required to establish definitively the role of ghrelin and other gut hormones in post-operative surgical patients.

## Abbreviations

CABG, coronary artery bypass grafting

MAC, mid arm circumference

VAS, visual analogue scale

## Competing interests

The author(s) declare that they have no competing interests.

## Authors' contributions

GF, AB, SJB, PIH and MN were responsible for designing the study. GF, AB, SJB, and PIH conceived the study, and supervised the data collection and analysis. MN and MP performed the statistical analysis. SRB, MG and MP contributed for radioimmunoassays, and interpretation of ghrelin results. MN was responsible for recruiting CABG patients and control subjects, taking blood, postprandial, data collections and analysis, undertaking radioimmunoassays, and manuscript preparation. All authors read and approved the final manuscript.
